# Magnetic Vestibular Stimulation (MVS) As a Technique for Understanding the Normal and Diseased Labyrinth

**DOI:** 10.3389/fneur.2017.00122

**Published:** 2017-04-05

**Authors:** Bryan K. Ward, Jorge Otero-Millan, Prem Jareonsettasin, Michael C. Schubert, Dale C. Roberts, David S. Zee

**Affiliations:** ^1^Department of Otolaryngology-Head and Neck Surgery, The Johns Hopkins University, Baltimore, MD, USA; ^2^Department of Neurology, The Johns Hopkins University, Baltimore, MD, USA; ^3^Department of Neuroscience, Exeter College, University of Oxford, Oxford, UK; ^4^Department of Physical Medicine and Rehabilitation, The Johns Hopkins University, Baltimore, MD, USA; ^5^Department of Neuroscience, The Johns Hopkins University, Baltimore, MD, USA; ^6^Department of Ophthalmology, The Johns Hopkins University, Baltimore, MD, USA

**Keywords:** vestibular diseases, dizziness, magnetic resonance imaging, labyrinthine fluids, magnetic vestibular stimulation, biophysics

## Abstract

Humans often experience dizziness and vertigo around strong static magnetic fields such as those present in an MRI scanner. Recent evidence supports the idea that this effect is the result of inner ear vestibular stimulation and that the mechanism is a magnetohydrodynamic force (Lorentz force) that is generated by the interactions between normal ionic currents in the inner ear endolymph and the strong static magnetic field of MRI machines. While in the MRI, the Lorentz force displaces the cupula of the lateral and anterior semicircular canals, as if the head was rotating with a constant acceleration. If a human subject’s eye movements are recorded when they are in darkness in an MRI machine (i.e., without fixation), there is a persistent nystagmus that diminishes but does not completely disappear over time. When the person exits the magnetic field, there is a transient aftereffect (nystagmus beating in the opposite direction) that reflects adaptation that occurred in the MRI. This magnetic vestibular stimulation (MVS) is a useful technique for exploring set-point adaptation, the process by which the brain adapts to a change in its environment, which in this case is vestibular imbalance. Here, we review the mechanism of MVS, how MVS produces a unique stimulus to the labyrinth that allows us to explore set-point adaptation, and how this technique might apply to the understanding and treatment of vestibular and other neurological disorders.

In 2009, Marcelli et al. were studying the effects of a caloric (cold water) vestibular stimulus on the brain using functional MRI (fMRI) when they observed a slow drift of the eyes in some of their subjects while they lay in darkness inside the 1.5 T magnetic field, *before* irrigating the ear or taking any images (1). Recall that, in an MRI machine, the large static magnetic field is always active, whereas high frequency oscillations in the magnetic field are only present when obtaining images. Marcelli et al. speculated that this unexpected nystagmus in the MRI machine was due to effects of the strong static magnetic fields on the labyrinth itself. Key to making this observation was the elimination of the visual fixation mechanisms that normally suppress unwanted nystagmus induced from an imbalance in labyrinthine inputs. Bringing out or increasing the intensity of a nystagmus by removal of visual fixation is a classical technique in the examination of patients with vestibular disorders. It helps one to decide if the lesion might be in central pathways in the brain, in which case fixation often has no effect on the intensity of nystagmus. This maneuver is the equivalent in the vestibulo-ocular system of the Romberg sign of classical neurology in which eliminating vision causes patients to lose balance if the contributions of their vestibular or somatosensory systems to postural stability are deficient. By eliminating visual fixation and relying on principles of the physical examination, Marcelli et al. had observed a direct effect of strong magnetic fields on the human vestibular system.

## Magnetic Vestibular Stimulation (MVS)—Discovering the Mechanism

While it had been known for decades that human subjects exposed to high-strength magnetic fields often feel off balance or dizzy (2–6), the observation by Marcelli et al. was the first objective sign of a vestibular imbalance in human subjects that were inside high-strength MRI machines. Prior animal experiments, in which the effects of strong magnetic fields on posture were studied (7–10), suggested that stimulation of the labyrinth might be the culprit but not enough evidence had been marshaled to develop a convincing explanation of the actual mechanism.

**Figure 1:**
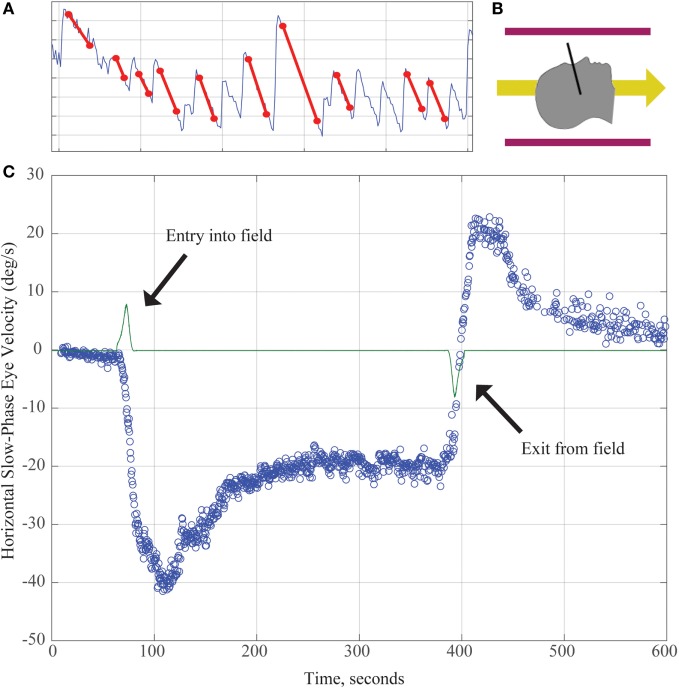
**(A)** Horizontal position of the eye as a function of time demonstrating slow and quick phases of nystagmus over about 8 s. The slope of the slow component (marked with red lines) is used to calculate the slow-phase velocity (SPV) in panel **(C)**. **(B)** Subjects enter the magnetic field head-first (magnetic field vector shown by yellow arrow). Black line demonstrates approximate location of Reid’s plane. **(C)** SPV versus time. The green line represents electromagnetic induction due to motion through the magnetic field gradient, with peaks marking time of entry into/exit from the magnetic field. Each blue dot represents SPV of a beat of nystagmus. SPV increases to a peak shortly after entry before partially adapting. After exiting the magnet, an aftereffect appears with nystagmus in the opposite direction. Its rate of decay reflects the dissipation of the adaptation that had taken place inside the MRI.

**Figure 2:**
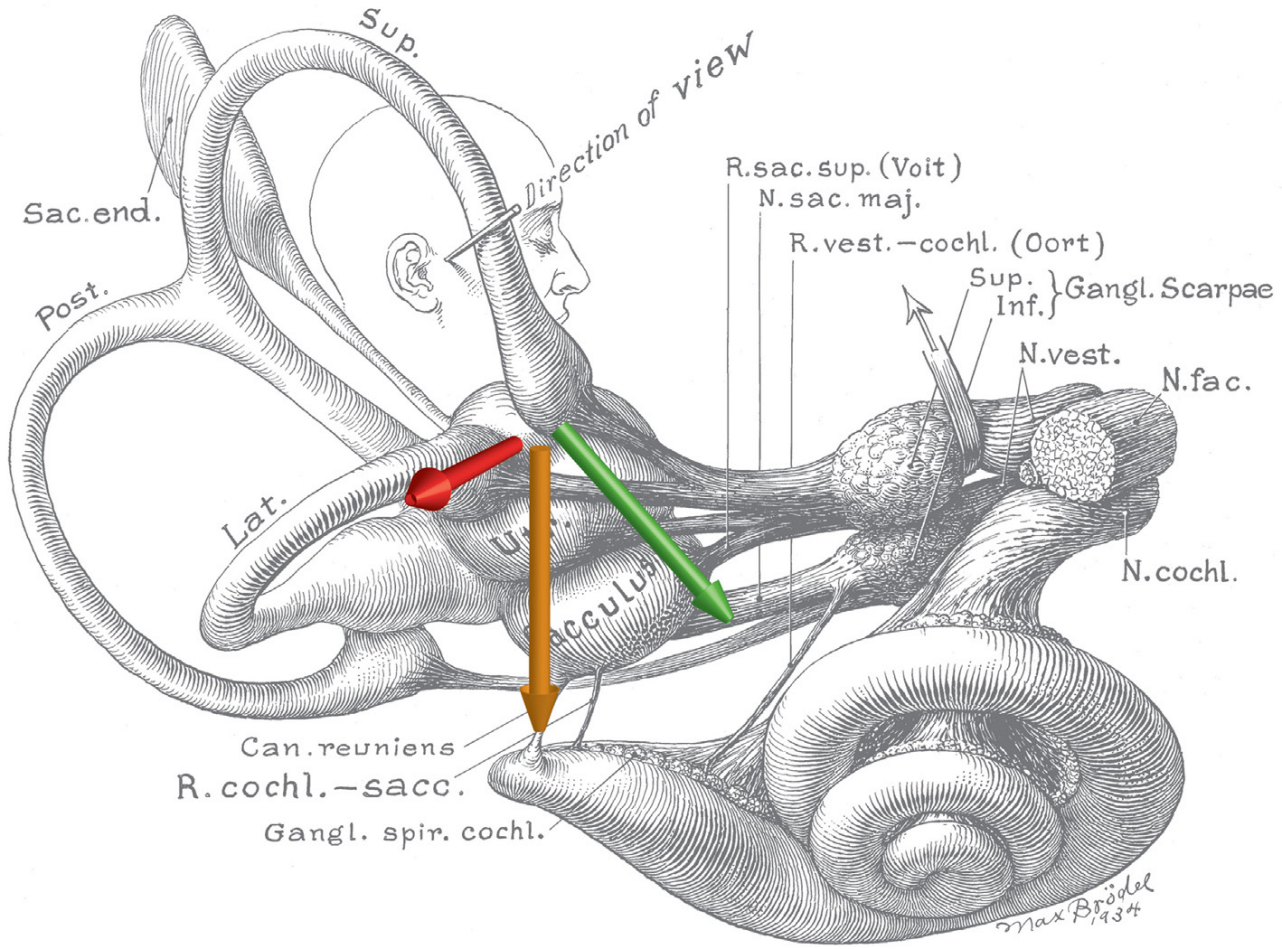
**Schematic of the inner ear with approximate locations of magnetic field, net ionic current, and Lorentz force vectors**. Note the anatomic relationship between the utricle and ampulla of the lateral and anterior semicircular canals. The green arrow represents the net ionic current of the utricle. The orange arrow represents the direction of the magnetic field vector (head to toe), when a person is lying in the strong static magnetic field of an MRI. The red arrow represents the resultant net Lorentz force from the cross product of the current and magnetic field vectors. Original illustration #933 in the Walters Collection of the Max Brödel Archives, Department of Art as Applied to Medicine, The Johns Hopkins University School of Medicine, Baltimore.

Working with Marcelli, we investigated this phenomenon further by recording eye movements in normal humans exposed to a strong (7 T) static magnetic field (11). No images were taken so that the effect could be attributed entirely to the constant magnetic field of the MRI machine. With elimination of visual fixation and recording eye movements with infrared video techniques, we found that (1) all normal human subjects tested had horizontal nystagmus while lying in the MRI bore (Figure 1); (2) the direction of nystagmus reversed with extreme head pitch, i.e., there was a null position in which there was no nystagmus; (3) the direction of nystagmus reversed with direction of entry (feet first versus head first into the front of the bore or head first into the front of the bore versus head first into the back of the bore); (4) the effect showed some decay while the subject was in the bore, but nystagmus was still present at 90 min of stimulation, the longest time tested; (5) the effect did not depend on rate of motion into or out of the bore; (6) the intensity of the nystagmus scaled roughly linearly with the strength of the magnetic field (comparing responses in a 7- and 3-T magnet); and (7) no nystagmus was observed in patients with bilateral peripheral vestibular loss, who were tested in several head positions to make sure being in a null orientation was not the reason for the absence of nystagmus (11).

Based on these compelling empirical observations and knowledge of magnetic field properties, we were able to rule out several candidate theories and hypothesize that the nystagmus arose because of a sustained pressure, transmitted *via* the endolymph fluid of the lateral semicircular canals, onto the cupula of the lateral semicircular canal. With this hypothesis, we presume that the pressure is generated by magnetohydrodynamic forces known as Lorentz forces arising from the interaction between the static magnetic field of the MRI machine and the constant mechano-electrical transduction currents in the ion-rich endolymphatic fluid. These ionic currents normally flow through the transduction channels of utricular hair cells and support their resting discharge rate. The Lorentz force, which is perpendicular to both the magnetic field and electrical current directions (Figure 2), can then generate a fluid pressure across the utricle and through the canal, producing a sustained displacement of the cupula, such as that which happens during a natural rotation of the head at a *constant acceleration*. The intensity of the nystagmus produced in the 7 T MRI machine was compatible with the known number of hair cells and the strength of their respective ion currents as well as the amount of fluid pressure and cupula displacement needed to produce a nystagmus of that intensity (11). Because one lateral canal is excited and the other is inhibited, the pattern of horizontal nystagmus is similar to that elicited by a natural rotation around the earth-vertical axis, with the exception of an additional torsional component in the MRI machine (see below). We emphasize here that the endolymph serves a dual purpose: it transmits ionic current into hair cells in the utricle to sustain their resting discharge, and it is the conduit by which the Lorentz force is transmitted as a pressure onto the cupula, pushing it to a persistently deviated position, producing a sustained nystagmus.

Further evidence from human patients with loss of function in one labyrinth indicates that there is also activation of the nearby anterior semicircular canals in the MRI machine, which in an intact subject would be excitation of one canal and inhibition of the other (12). Based on the classical studies of the nineteenth century giants of vestibular physiology—Ewald, Flourens, and Breuer—we predicted that, in intact subjects, any vertical components to the nystagmus from activation of the anterior semicircular canals would subtract, but any torsional components (because they are oppositely directed) would add. Recall Ewald’s laws, (1) activation of an individual canal induces eye (or head) motion in a plane parallel to that of the activated semicircular canal, (2) the lateral SCC have a brisker response to ampullopetal (excitation) than ampullofugal (inhibition) fluid flow, and (3) the vertical SCC have a brisker response to ampullofugal (excitation) than ampullopetal (inhibition) fluid flow. When only one labyrinth is functioning, there will still be a horizontal component of the nystagmus (either from excitation or inhibition of the remaining lateral SCC) but also a vertical component, the direction depending on which anterior canal remains intact. Considering how the magnetic field is oriented in our scanner (field pointing head to toe), most normal subjects develop a horizontal nystagmus with slow phases to the left because the right lateral canal is being excited and the left inhibited. In patients with a loss of labyrinthine function on the left side, the unbalanced anterior canal inputs lead to an upward beating component to their nystagmus while patients with loss on the right side develop a downward beating component to their nystagmus (12). Since activation of an anterior semicircular canal also induces torsion, in intact human subjects not only should the vertical components cancel but the torsional components (which are in opposite directions in the two labyrinths) should sum since one side is being inhibited and the other excited. This produces a torsional component that adds to the horizontal component. This indeed is the case so that normal human subjects show a mixed horizontal–torsional nystagmus in the MRI machine (see Video S1 in Supplementary Material). The pattern is unusual, however, in that the top poles of the eyes rotate in a slow phase *away* from the ear toward which the horizontal component of the slow phase is directed. In contrast, in the more common pattern of a mixed horizontal–torsional nystagmus, for example, as seen after a unilateral loss of labyrinthine function, the top poles of the eyes rotate in a slow phase *toward* the same ear as the horizontal component of the slow phase is directed (13). We emphasize here that these seemingly complicated patterns of nystagmus are easily derived from the laws of Ewald and Flourens, known for nearly 150 years.

One other issue that was raised in our initial study was why there was so much variability in the null position among subjects (range up to 50°) (11). We can only speculate, but we attribute some portion to the inherent variability of the orientation of the bony labyrinth in the skull and the rest to other natural physiological and geometric variation of the labyrinth. The implications of a null are important since fMRI studies of all types, including resting-state connectivity become susceptible to artifacts from stimulation of the labyrinth by the magnetic field, and in turn, the brain. Whether fixation is prevented (the eyes are closed or the subject is in darkness), or the eyes are open, allowing fixation suppression of the induced nystagmus, the brain will be activated in many regions. Note there are wide spread projections of vestibular sensory information to almost every area of the brain (14, 15). This vestibular activation can introduce a confound when trying to relate behaviors to changes in the metabolic activity in the brain. Of course, this problem goes away if the head of the subject is positioned in the scanner in the null position where no nystagmus is produced.

## MVS: An Experimental Paradigm for “Set-Point Adaptation”

### How Does the Brain Keep Central Tone Balanced?

Recognized by Sherrington as a key principle of postural control, tone (set point) is optimally achieved *via* sustained balance level of opposing tonic neural activity in a push–pull fashion (16, 17). Semicircular canals work in coplanar pairs that are reciprocally excited and inhibited as they transduce head rotations, therefore, serve as a prototypical model for the study of set-point adaptation.

Consider the archetypical clinical challenge when a human suddenly loses function of one labyrinth: a strong spontaneous nystagmus initially appears due to the central tone imbalance that is created by the asymmetry in afferent activity from the labyrinths. The nystagmus eventually subsides as central balance is restored, but usually takes days to weeks to completely resolve (18) Furthermore, should a patient who has recovered from a previous loss of function in one labyrinth suddenly lose function in the remaining labyrinth, the patient shows a strong spontaneous nystagmus similar to but oppositely directed from that brought on by the original loss of function on one side (19). This “Becheterew’s phenomenon” occurs even when neither labyrinth is active as shown by absent caloric responses. The reason, of course, is that activity within the vestibular nuclei had been rebalanced after the initial loss of function in one labyrinth. When activity from the remaining labyrinth is subsequently lost, a new imbalance is created centrally just as if the function of a single labyrinth was removed again. This sequence of events simply reflects the ability of the brain to rebalance activity centrally in spite of a persistent asymmetry in peripheral activity.

Without adaptation (or what some call vestibular compensation), persistent nystagmus impairs vision, thus the brain must have mechanisms to rid itself of nystagmus. How does this happen?

### Early Studies of Set-Point Adaptation

Nearly 50 years ago, seminal studies were performed almost simultaneously by two groups addressing the mechanisms by which nystagmus is removed when there is a sustained vestibular imbalance (18, 20). Normal human subjects rotated in a chair at *a constant acceleration* develop a nystagmus that is sustained because of the mechanical properties of the cupula-semicircular canal and endolymph. Any relatively constant sustained nystagmus is a low-frequency stimulus and is presumably interpreted by the brain as resulting from disease since sustained nystagmus almost never occurs naturally unless there is a lesion producing a persistent tone imbalance. Of course, this experimental paradigm using sustained rotation of the head, while producing a sustained nystagmus, does not perfectly mimic a naturally occurring lesion in one labyrinth because in normal subjects there is a tonic level of activity coming from both labyrinths and one side is excited and the other inhibited. Nevertheless, by using a constant acceleration stimulus, important information was gleaned about the immediate response of the brain to a vestibular imbalance. By its nature, however, a constantly accelerating head rotation can only be imposed so long before the speed of rotation exceeds values that are feasible or safe. Consequently, during rotation, a sustained nystagmus could only be maintained for a few minutes so that any adaptation was incomplete and relatively short-lived with decay time constants on the order of few minutes. Nevertheless, quantitative models were developed that nicely mimicked the real data, though nothing could be inferred about adaptation taking place beyond the first few minutes of a sustained vestibular imbalance.

A sustained nystagmus can also be elicited with prolonged caloric irrigation of the ear canal. Several groups observed that a long-duration caloric irrigation appeared to reach a steady state (up to 60 min), as the nystagmus did not decay to 0 (21, 22). Prolonged caloric stimulation, however, is uncomfortable and also variable among individuals depending upon the thermal conductivity and anatomical configuration of the bony labyrinth in the skull. While the main effect of caloric stimulation is changing the density of the fluid within the semicircular canal, it also has a direct thermal effect on nervous tissue. Even so, the persistent nystagmus from a caloric irrigation appears to be independent of any direct thermal effect on activity in the hair cells and the vestibular nerve (22). In spite of these limitations, a caloric stimulus has one advantage over MVS since it is unilateral and, in this way, better mimics a lesion of one lateral semicircular canal. MVS, while producing a sustained nystagmus, excites one lateral semicircular canal and inhibits the other.

### MVS to Elicit “Set-Point Adaptation”

Recently, we used MVS to extend our knowledge of the time courses of rebalancing tone within the vestibular nuclei, as a model of set-point adaptation. Magnetic vestibular stimulation (MVS) is a simple, safe, and comfortable way to induce a sustained nystagmus and is uniquely suited to study how the brain adapts to a sustained vestibular imbalance. We placed subjects in a 7 T MRI for up to 90 min, during which they showed a persistent nystagmus (23). During this period of sustained MVS, however, slow-phase velocity, after rising relatively quickly to its maximum value, slowly began to decay. We assume that if the MVS could have been sustained for even longer periods, the nystagmus would have eventually disappeared, as occurs over days following an actual loss of function, e.g., after a vestibular nerve section. This gradual fading of the MVS nystagmus represents the action of adaptive processes that monitor and adjust set points. When the adaptive stimulus is abruptly removed, as when the subject exits the MRI bore, an aftereffect appears, revealing the prior adaptation, with oppositely directed slow phases that slowly dissipate at a rate commensurate with how long the initial nystagmus was sustained in the magnet.

**Figure 3:**
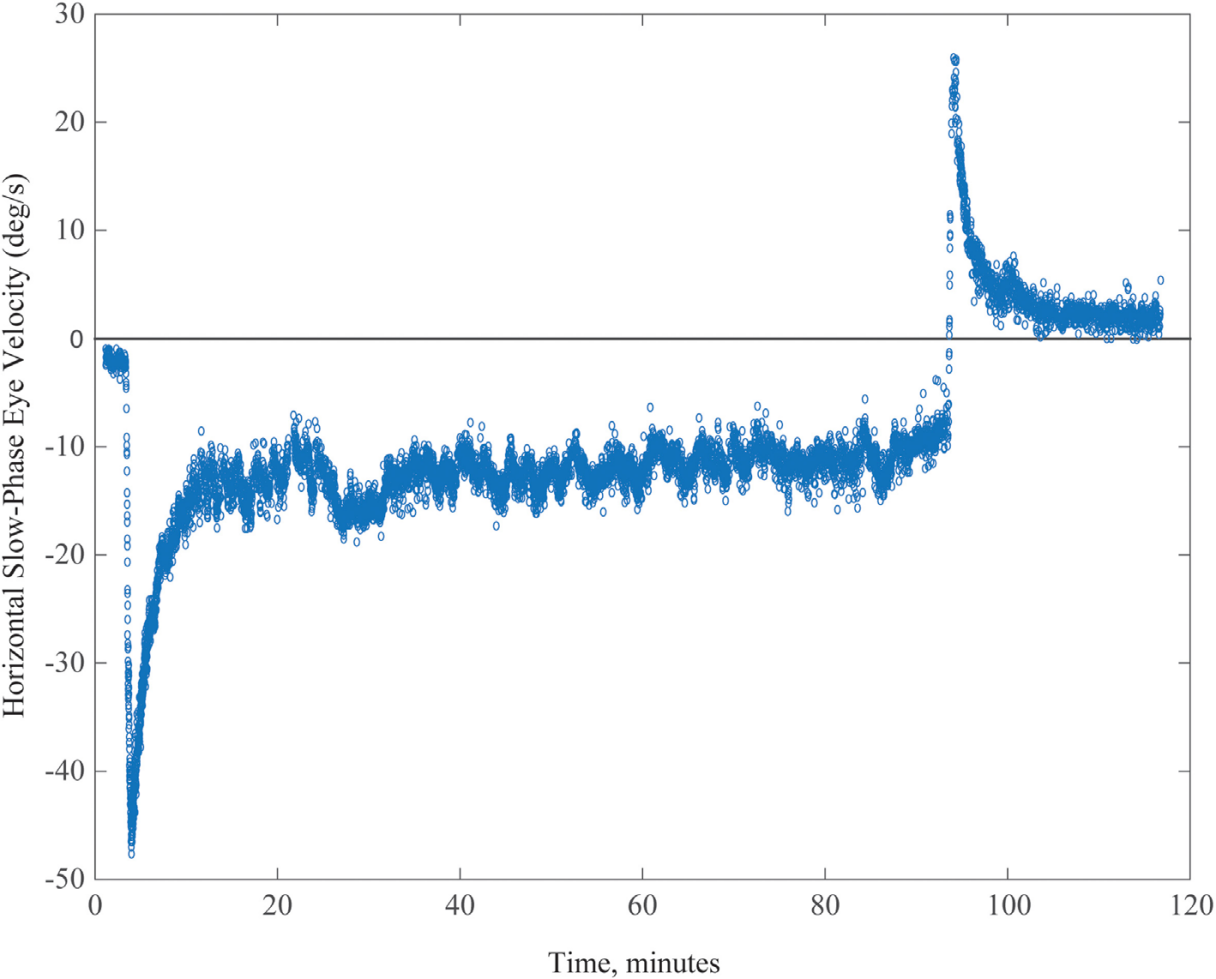
**Example of spontaneous nystagmus for up to 90 min in an MRI machine**. Graph shows horizontal slow-phase eye velocity of a single subject with negative representing left and positive right. The nystagmus incompletely adapts, and the aftereffect exceeds 30 min, reflecting the relatively long duration of being in the MRI machine.

A typical example of MVS-induced nystagmus and the attempt of an adaptive mechanism to rid itself of it is shown in Figure 3 (data points) for a 90-min period of stimulation. Our observations suggest a process that occurs over multiple time courses, and we developed an analytical model that incorporated three integrators of varying dynamic properties (progressively longer time constants) to simulate our data (23). Slower dynamic properties shift the system in stages toward a new 0 set point by gradually eliminating the unwanted bias.

### Error Signals and the Implementation of the Adaptive Response

**Figure 4:**
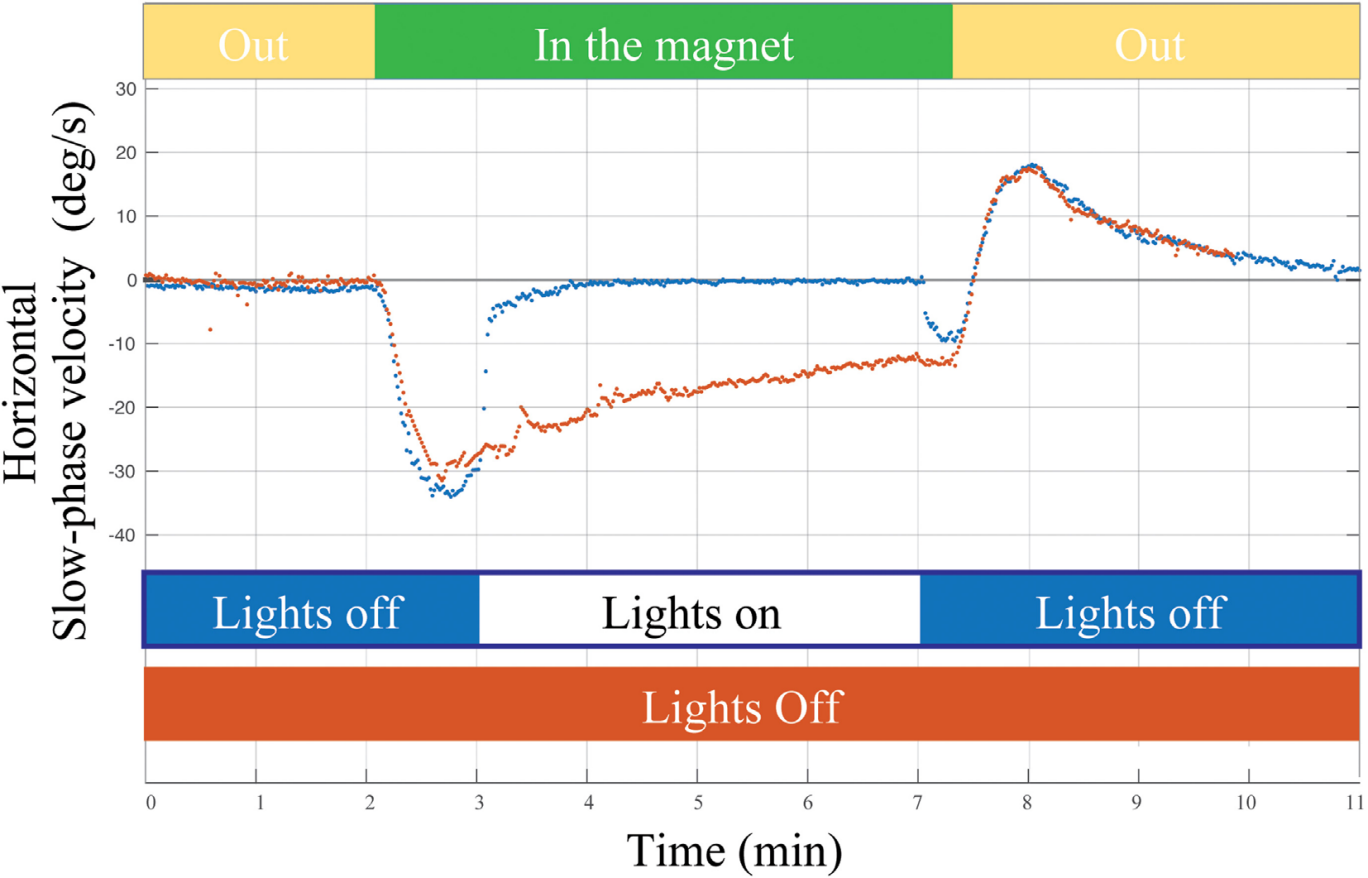
**Example of a single subject entering the MRI head first supine position in two conditions: (1) lights off for 2 min outside the magnet, 5 min inside the magnet, and 2 min outside the magnet (orange trace) and (2) lights turned on for approximately 4 min of fixation inside the magnet (blue trace)**. Visual fixation had negligible impact on the rate of adaptation or on the aftereffect (reflecting adaptation in the MRI regardless of whether the lights were on or off).

What are the error signals responsible for driving set-point adaptation of the vestibulo-ocular reflex (VOR)? Although vision and retinal slip are critical signals for adaptation of the dynamic components of the VOR, i.e., the gain of the VOR during head movement, they appear to be unnecessary or at least have only a minimal impact upon VOR set-point adaptation. As a consequence of spontaneous nystagmus, retinal slip is an obvious error signal to drive set-point adaptation. We found, however, that the rate of the early components of adaptation in the MRI scanner, and their aftereffect (reflecting the adaptive rebalancing that had occurred while in the MRI scanner) were nearly superimposable regardless of whether the eyes were fixing on a target or the subject was in darkness in the MRI machine (Figure 4). Whether or not vision influences the later components of set-point adaptation is unknown, but after acute unilateral labyrinthectomy in the monkey, an absence of vision does not prevent the disappearance of the spontaneous nystagmus (24).

Where in the brain do these processes of vestibular adaptation take place? For dynamic adaptation of the gain of an inappropriate VOR, signals of unwanted retinal image motion during head movements are thought to be received by the cerebellum that, in turn, recalibrates the eye movement response. For static, set-point adaptation of the VOR, there must exist a mechanism that monitors the spontaneous neural discharge occurring at the vestibular nuclei in order to rebalance the activity between the two sides. The commissural system between the vestibular nuclei may mediate the rebalancing of activity (25, 26). Whether the cerebellum is involved in this process is uncertain (27), but there is evidence from other learning paradigms that the cerebellum participates in set-point adaptation. Examples included control of posture, pointing, and alignment of the eyes (28–30). Furthermore, it is unknown how the brain implements set-point adaptation with multiple time constants, but as in vestibular compensation, this is likely to occur at multiple biological levels—changes in gene expression, neurotransmitter levels, inflammatory responses, synaptic activity, neurogenesis, and other unidentified changes at cellular, network, and cognitive levels (31). Future knowledge on the substrates of adaptation may lead to ways to alter the time constants favorably, allowing new treatments for human disease.

## Further Implications and Future Directions of MVS

Although our interest in MVS began as a curiosity—why were people experiencing dizziness in a strong MRI?—an understanding of the mechanism has greatly expanded its applicability. MVS has helped us to understand how the brain restores equilibrium in response to a sustained vestibular imbalance, such as which occurs in the more natural circumstance when one labyrinth is impaired. These studies have implications for how the brain adapts to any environmental perturbation or unwanted internal change that requires a change in set point to maintain equilibrium and balanced tone and may offer clues to the pathophysiology of set-point disorders such as ocular motor integrator defects producing nystagmus and cervical dystonia producing wry neck. In the vestibular system, we can now explore which parts of the brain are involved in the various time courses of adaptation.

There are implications too for studies of functional and resting-state MRI. On the caveat side, any induced nystagmus, whether or not it is suppressed by fixation, will be accompanied by dramatic changes in activity in many parts of the brain due to the widespread projections of information emanating from the labyrinth. Such metabolic activity could be an important confound in interpreting fMRI studies (32, 33) as well as the fact that vestibular activation of MVS is subject to adaptive mechanisms that try and eliminate it. Positioning the head in the null position might eliminate the MVS effect. On the other hand, MVS-induced nystagmus and the corresponding changes in metabolic activity in the brain can be used to map out the central projections of vestibular afferent activity [e.g., Hitier et al. (34)] [as Marcelli et al., tried to do with his original studies using caloric stimuli when MVS was discovered (1)] as well as changes in activity in different locations that reflect vestibular adaptation over the different time courses that we have discovered.

We also foresee applications to clinical problems. By studying patients with vestibular disorders, we may learn how and in which structures the brain has adapted to a prior lesion, and how it might adapt to a new vestibular imbalance, be it static (involving resting tone) or dynamic (involving compensatory movements in response to head rotations). One wonders if persistent MVS might be a form of vestibular therapy itself, prodding the brain to adapt better to previous vestibular disorders. Furthermore, in a stationary, supine subject, MVS creates an unnatural pattern of labyrinthine activation. The semicircular canal activation signals the head is rotating but the otoliths signals do not since the head is stationary and the gravity vector is unchanging. Recall when a subject is rotating around an Earth-horizontal axis while lying parallel to the ground (barbecue rotation), there will be a changing gravity vector that activates the otoliths that also signals (like the accompanying canal activation) that the head is rotating. Thus, the “unnatural” pattern of activation in the MRI machine may be used to explore the pathophysiology of the disordered and often disabling perceptions in patients in whom the problem seems to be an inability to resolve conflicts among visual, proprioceptive, and vestibular information. Even higher-level cognitive disorders, e.g., hemi-spatial neglect, might be helped by MVS. Such disorders have been shown previously to be temporarily ameliorated using caloric or galvanic stimulation (35, 36), but these are cruder and less easily tolerated forms of labyrinthine activation.

To epitomize, MVS is a relatively simple, safe, and novel way of stimulating the labyrinth. Much as did caloric, galvanic, and artificial rotational stimulation, we expect that MVS will bring important scientific and clinical advances to our understanding of how the vestibular system works and how it repairs itself when faced with disease or trauma.

## Author Contributions

DZ and BW performed the initial drafts of the manuscript. DR created the initial draft of Figure 1. BW created Figure 2. JO-M created the initial versions of Figures 3 and 4. All authors were involved in data collection and editing and approval of the final version of the manuscript.

## Conflict of Interest Statement

The authors declare that the research was conducted in the absence of any commercial or financial relationships that could be construed as a potential conflict of interest.

## Supplementary Material

Video S1 **A mixed horizontal–torsional eye movement in a healthy adult lying supine in the bore of a 7-T MRI machine**. This unusual nystagmus pattern has a torsional component in which the top poles of the eyes rotate in a slow phase pattern of nystagmus away from the ear toward which the horizontal component of the slow phase is directed. This is in contrast to the more common pattern of a mixed horizontal–torsional nystagmus, for example, as seen after a unilateral loss of labyrinthine function, where the top poles of the eyes rotate in a slow phase toward the same ear as the horizontal component of the slow phase is directed. This reflects the unusual combined stimulation pattern of the anterior and lateral semicircular canals.
